# Cytotoxic and Antibacterial Cembranoids from a South China Sea Soft Coral, *Lobophytum* sp

**DOI:** 10.3390/md11041162

**Published:** 2013-04-03

**Authors:** Min Zhao, Jian Yin, Wei Jiang, Minshan Ma, Xinxiang Lei, Zheng Xiang, Jianyong Dong, Kexin Huang, Pengcheng Yan

**Affiliations:** 1 School of Pharmacy, Wenzhou Medical College, Wenzhou 325035, China; E-Mails: miniezhao@gmail.com (M.Z.); yinjian198708@gmail.com (J.Y.); xzh007@126.com (Z.X.); jianyd@wzmc.edu.cn (J.D.); 2 College of Environmental Science and Engineering, Yangzhou University, Yangzhou 225127, China; E-Mail: weijiang@yzu.edu.cn; 3 Analytical and Testing Center, Wenzhou University, Wenzhou 325035, China; E-Mails: youihj1023@gmail.com (M.M.); xxlei@wzu.edu.cn (X.L.)

**Keywords:** soft coral, *Lobophytum*, cembranoids, cytotoxicity, antibacterial activity

## Abstract

Chemical examination of a South China Sea soft coral *Lobophytum* sp. led to the isolation of three new α-methylene-γ-lactone-containing cembranoids, (1R*,3R*,4R*,14R*,7*E*,11*E*)-3,4-epoxycembra-7,11,15(17)-trien-16,14-olide (**1**), (1R*,7S*,14S*,3*E*,11*E*)-7-hydroperoxycembra-3,8(19),11,15(17)-tetraen-16,14-olide (**2**), and (1R*,7S*,14S*,3*E*,11*E*)-18-acetoxy-7-hydroperoxycembra-3,8(19),11,15(17)-tetraen-16,14-olide (**3**), along with eleven known analogues **4**–**14**. The structures of the new compounds were elucidated through extensive spectroscopic analysis, including 1D and 2D NMR data. Compounds **1**–**3** exhibited moderate cytotoxic activity against the selected tumor cell lines. Moreover, **2** and **3** were found to be moderate inhibitors against the bacteria *S. aureus* and *S. pneumoniae*.

## 1. Introduction

**Figure 1:**
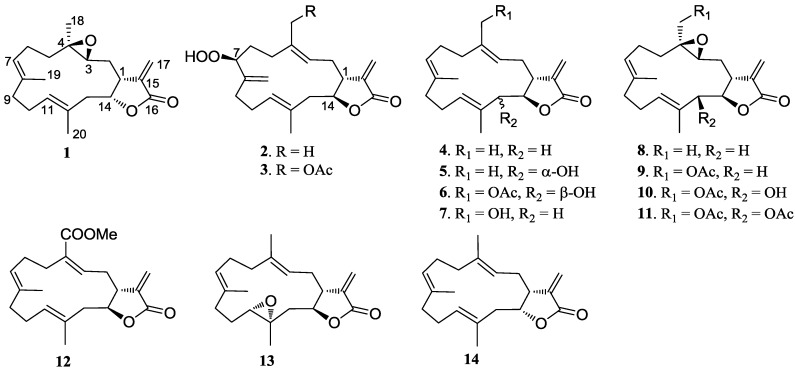
Structures of compounds **1**–**14**.

Soft corals belonging to the genus *Lobophytum* (Alcyoniidae) have been shown to be a rich source of macrocyclic cembranoids and their cyclized derivatives [1,2,3,4,5,6,7,8,9,10,11,12,13,14], commonly described as defensive substances against predators such as other corals and fishes [15,16]. Some of these metabolites are of considerable interest and merit continuous attention due to their unique structures and significant biological activities, including anti-tumor, anti-viral, and anti-inflammatory properties [1,2,3,4,5,6,7,8,9,10,11,12,13,14]. As part of our ongoing research on bioactive marine natural products from *Lobophytum* corals of South China Sea [17,18,19,20], a Hainan soft coral, *Lobophytum* sp., has been chemically investigated based on the EtOAc extracts showing cytotoxicity against a panel of tumor cell lines including SGC7901 (human gastric carcinoma), A549 (human lung epithelial carcinoma), MCF7 (human breast carcinoma), HCT116 (human colonic carcinoma), and B16 (mouse melanoma). Chemical investigation resulted in the isolation of three new α-methylene-γ-lactone-containing cembranoids (**1**–**3**), along with eleven known analogues **4**–**14** (Figure 1). The compounds isolated were evaluated for their cytotoxicity against selected tumor cell lines and antimicrobial activity. This paper deals with the isolation, structural elucidation, and bioactivity of these compounds.

## 2. Results and Discussion

Repeated column chromatography of the EtOAc fraction of the soft coral *Lobophytum* sp. resulted in the isolation and characterization of three new α-methylene-γ-lactone-containing cembranoid diterpenes, namely (1R*,3R*,4R*,14R*,7*E*,11*E*)-3,4-epoxycembra-7,11,15(17)-trien-16,14-olide (**1**), (1R*,7S*,14S*,3*E*,11*E*)-7-hydroperoxycembra-3,8(19),11,15(17)-tetraen-16,14-olide (**2**), and (1R*,7S*,14S*,3*E*,11*E*)-18-acetoxy-7-hydroperoxycembra-3,8(19),11,15(17)-tetraen-16,14-olide (**3**), along with eleven known related analogues **4**–**14**. The structures of known compounds were identified by analysis of the NMR spectroscopic data and by comparison with those reported in the literature. They were identified as (1R*,14S*,3*E*,7*E*,11*E*)-cembra-3,7,11,15(17)-tetraen-16,14-olide (**4**) [21], lobophytolide E (**5**) [22], durumolide B (**6**) [3], 13-dehydroxylpresinularolide B (**7**) [4], isolobophytolide (**8**) [21], lobolide (**9**) [23], 13-hydroxylobolide (**10**) [23], 13,18-diacetylsinularolide B (**11**) [4], lobophytolide B (**12**) [22], lobophytolide (**13**) [24], and lobophytolide A (**14**) [22], respectively. It is worthy to point out that 13,18-diacetylsinularolide B (**11**) [4], previously obtained by acetylation of 13-hydroxylobolide (**10**), is reported herein as a natural product for the first time.

**Figure 2:**
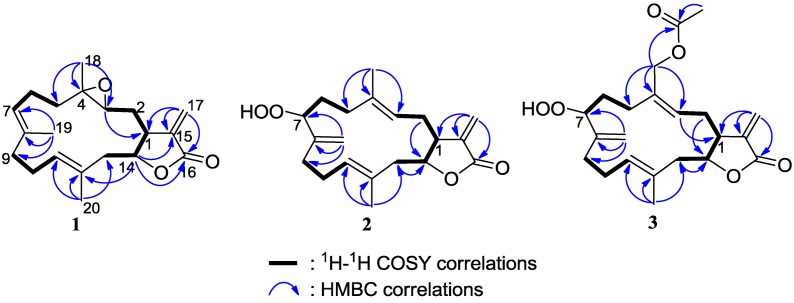
COSY and HMBC correlations of compounds **1**–**3**.

**Figure 3:**
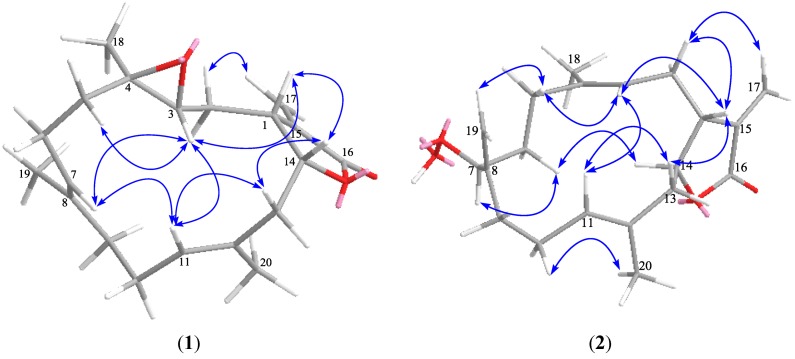
Key NOE correlations and computer-generated models using MM2 force field calculations for compounds **1** and **2**.

Compound **1** was obtained as a colorless oil. The molecular formula, C_20_H_28_O_3_, consistent with seven degrees of unsaturation, was established by HRESIMS *m*/*z* 317.2105 [M + H]^+^ (Calcd. 317.2111) and NMR data. The ^1^H NMR spectrum of **1** exhibited the signals for three methyl groups including two olefinic methyls at δ_H_ 1.74 (3H, s, H_3_-20) and 1.62 (3H, s, H_3_-19), and a tertiary methyl at δ_H_ 1.27 (3H, s, H_3_-18), while the ^13^C NMR spectrum displayed 20 carbon resonances including a carbonyl and six olefinic carbons (Table 1, Table 2). IR absorptions at 1759 and 1660 cm^−^^1^ suggested the presence of an α-methylene-γ-lactone group [3,21]. This assumption was further supported by the ^1^H NMR signals at δ_H_ 6.26 (1H, d, *J* = 2.4 Hz, H-17a), 5.52 (1H, d, *J* = 2.4 Hz, H-17b), and ^13^C NMR signals at δ_C_ 169.9 (C, C-16), 138.3 (C, C-15), 120.8 (CH_2_, C-17), 77.1 (CH, C-14), and 42.6 (CH, C-1). Four olefinic carbon signals at δ_C_ 135.1 (C, C-8), 130.1 (CH, C-11), 129.6 (C, C-12), and 122.3 (CH, C-7), and two olefinic proton signals at δ_H_ 5.11 (1H, br d, *J* = 9.0 Hz, H-11) and 4.95 (1H, dd, *J* = 7.2, 1.8 Hz, H-7) were attributed to two trisubstituted double bonds. In addition, a trisubstituted epoxide was observed from carbon signals at δ_C_ 62.3 (CH, C-3) and 60.8 (C, C-4), as well as an oxymethine proton at δ_H_ 2.58 (1H, dd, *J* = 10.2, 1.2 Hz, H-3). Six degrees of unsaturation, accounted for by the functional groups from seven in the molecule, suggested the remaining of a cyclic structure in **1**. By interpretation of ^1^H–^1^H COSY correlations, three partial structures extending from H_2_-13 to H-3, from H_2_-5 to H-7, and from H_2_-9 to H-11 were established. Moreover, the connectivities of these partial structures were established by HMBC correlations and revealed a 14-membered cembrane-type diterpenoid skeleton for **1** (Figure 2). The location of the epoxide at C-3 and C-4 was indicated by the HMBC correlations observed from H_3_-18 to C-3, C-4, and C-5, while the locations of two double bonds at C-7/C-8 and C-11/C-12 were disclosed by the HMBC correlations from H_3_-19 to C-7, C-8, and C-9, and from H_3_-20 to C-11, C-12, and C-13. Thus, the gross structure of **1** was determined to be the same as isolobophytolide (**8**) [21], possessing an α-methylene-γ-lactone ring fused to a 14-membered ring at C-1 and C-14. However, the diagnostic upfield shift of C-2 (−6.3 ppm) due to the presence of the γ-gauche effect was observed in **1**, compared to **8**, suggesting the lactone ring at C-1 and C-14 was *cis*-fused. On the other hand, according to the general empirical rule that all cembrane diterpenes of known absolute configuration at C-1 reported from the order Alcyonacea belong to the α series [22], the configurations at C-1 and C-14 in **1** were assigned to be consistent with those of the co-occurring analogue lobophytolide A (**14**) [22]. The geometry of two double bonds at C-7/C-8 and C-11/C-12, and stereogenic centers at C-3 and C-4 were in agreement with those of isolobophytolide (**8**) on the basis of the similar NMR data. The stereochemistry of **1** as assigned above was further confirmed by the NOESY correlations (Figure 3) between H-1/H-14, H-1/H-3, H-3/H-5b, H-3/H-11, H-11/H-13b, and H-14/H-13b. Consequently, compound **1** was elucidated as a C-14 epimer of isolobophytolide (**8**), namely (1R*,3R*,4R*,14R*,7*E*,11*E*)-3,4-epoxycembra-7,11,15(17)-trien-16,14-olide.

**Table 1:** ^13^C NMR data for compounds **1**–**3** (CDCl_3_, 150 MHz).

No.	1, δ_C_, type	2, δ_C_, type	3, δ_C_, type
1	42.6, CH	45.1, CH	45.0, CH
2	26.0, CH_2_	31.7, CH_2_	32.1, CH_2_
3	62.3, CH	121.1, CH	127.4, CH
4	60.8, C	137.2, C	135.9, C
5	38.7, CH_2_	34.7, CH_2_	30.6, CH_2_
6	23.9, CH_2_	28.6, CH_2_	28.6, CH_2_
7	122.3, CH	84.5, CH	84.2, CH
8	135.1, C	149.2, C	149.0, C
9	38.1, CH_2_	33.5, CH_2_	33.4, CH_2_
10	25.0, CH_2_	28.4, CH_2_	28.5, CH_2_
11	130.1, CH	128.0, CH	128.4, CH
12	129.6, C	131.3, C	131.0, C
13	42.7, CH_2_	44.6, CH_2_	44.7, CH_2_
14	77.1, CH	81.7, CH	81.3, CH
15	138.3, C	139.2, C	138.9, C
16	169.9, C	170.2, C	169.9, C
17	120.8, CH_2_	121.9, CH_2_	122.5, CH_2_
18	16.5, CH_3_	16.3, CH_3_	61.9, CH_2_
19	16.2, CH_3_	111.6, CH_2_	111.9, CH_2_
20	15.8, CH_3_	17.5, CH_3_	17.6, CH_3_
–OAc	–	–	20.9, CH_3_
–OAc	–	–	171.0, C

**Table 2:** ^1^H NMR data for compounds **1**–**3** (CDCl_3_, 600 MHz).

No.	1, δ_H_ (*J* in Hz)	2, δ_H_ (*J* in Hz)	3, δ_H_ (*J* in Hz)
1	3.36, ddd (10.8, 7.2, 3.6)	2.79, m	2.81, m
2	a: 2.16, m	a: 2.33, m	
	b: 1.42, ddd	b: 2.25, m	2.41, m
	(14.4, 10.2, 3.6)		
3	2.58, dd (10.2, 1.2)	5.16, t (7.2)	5.43, t (7.2)
5	a: 2.11, m	a: 2.24, m	a: 2.34, m
	b: 1.14, m	b: 2.10, m	b: 2.22, m
6	a: 2.16, m	a: 1.76, m	a: 1.77, m
	b: 2.13, m	b: 1.67, m	b: 1.73, m
7	4.95, dd (7.2, 1.8)	4.35, dd (7.8, 4.2)	4.35, dd (8.4, 4.8)
9	a: 2.21, m	a: 2.34, m	a: 2.36, m
	b: 2.06, m	b: 2.00, m	b: 2.01, m
10	a: 2.39, m	a: 2.33, m	a: 2.26, m
	b: 2.10, m	b: 2.25, m	b: 2.24, m
11	5.11, br d (9.0)	5.23, t (7.2)	5.24, t (7.2)
13	a: 2.44, br d (14.4)	a: 2.47, dd (14.4, 4.8)	a: 2.49, dd (13.8, 4.2)
	b: 2.38, dd (14.4, 10.8)	b: 2.07, dd (14.4, 7.2)	b: 2.06, dd (13.8, 6.6)
14	4.97, m	4.30, m	4.33, m
17	a: 6.26, d (2.4)	a: 6.28, d (2.4)	a: 6.30, d (2.4)
	b: 5.52, d (2.4)	b: 5.63, d (2.4)	b: 5.67, d (2.4)
18	1.27, s	1.66, s	4.62, s
19	1.62, s	a: 5.13, br s	a: 5.13, br s
		b: 5.08, br s	b: 5.09, br s
20	1.74, s	1.67, s	1.68, s
–OAc	–	–	2.09, s
–OOH	–	7.78, br s	7.92, br s

Compound **2** was also obtained as a colorless oil. The molecular formula of **2** was determined to be C_20_H_28_O_4_ on the basis of HRESIMS data (*m*/*z* 355.1876 [M + Na]^+^, Calcd. 355.1880), implying seven degrees of unsaturation. Analysis of the ^1^H and ^13^C NMR spectra of **2** clearly revealed the presence of an α-methylene-γ-lactone moiety [δ_H_ 6.28 (1H, d, *J* = 2.4 Hz, H-17a), 5.63 (1H, d, *J* = 2.4 Hz, H-17b); δ_C_ 170.2 (C, C-16), 139.2 (C, C-15), 121.9 (CH_2_, C-17), 81.7 (CH, C-14), and 45.1 (CH, C-1)], two trisubstituted double bonds [δ_H_ 5.23 (1H, t, *J* = 7.2 Hz, H-11), 5.16 (1H, t, *J* = 7.2 Hz, H-3); δ_C_ 137.2 (C, C-4), 131.3 (C, C-12), 128.0 (CH, C-11), and 121.1 (CH, C-3)], an additional exocyclic double bond [δ_H_ 5.13 (1H, br s, H-19a), 5.08 (1H, br s, H-19b); δ_C_ 149.2 (C, C-8), 111.6 (CH_2_, C-19)], and two olefinic methyls [δ_H_ 1.67 (3H, s, H_3_-20), 1.66 (3H, s, H_3_-18); δ_C_ 17.5 (CH_3_, C-20), 16.3 (CH_3_, C-18)]. In addition, the presence of a hydroperoxyl group was disclosed by the NMR data at δ_H_ 4.35 (1H, dd, *J* = 7.8, 4.2 Hz, H-7), 7.78 (1H, br s, OOH) and δ_C_ 84.5 (CH, C-7), in association with the fact that two additional oxygen atoms remained in the molecule according to the HRESIMS data. All these data suggested that **2** possessed a cembrane skeleton with functionalities of an α-methylene-γ-lactone, two methyl-bearing trisubstituted double bonds, an exocyclic double bond, and a secondary hydroperoxyl group. Further interpretation of ^1^H–^1^H COSY and HMBC correlations established the gross structure of **2** as shown in Figure 2. The *E* geometry of two double bonds at C-3/C-4 and C-11/C-12, and the *trans* junction of the α-methylene-γ-lactone ring in **2** was determined based on the similar NMR data in comparison with those of the co-occurring analogue (1R*,14S*,3*E*,7*E*,11*E*)-cembra-3,7,11,15(17)-tetraen-16,14-olide (**4**) [21]. The configuration at C-7 was assigned by comparison of the ^13^C NMR chemical shift of C-7 with that of decaryiol D, a cembrane diterpene containing the same partial structure extending from C-5 to C-13 [25]. In decaryiol D, the absolute configuration at C-7 was determined as *R* unambiguously through structural transformation. While in compound **2**, significant upfield shift of C-7 (−6.7 ppm) was observed, compared to decaryiol D, allowing the assignment of C-7 S* in **2**. This assumption of stereochemistry for **2** was consistent with NOESY correlations (Figure 3), which indicated that H-1, H-3, H_2_-19, and H-11 were located on the same side of the ring system, whereas H-7 and H-14 were oriented toward the opposite side. Thus, compound **2** was defined as (1R*,7S*,14S*,3*E*,11*E*)-7-hydroperoxycembra-3,8(19),11,15(17)-tetraen-16,14-olide.

Compound **3** had a molecular formula of C_22_H_30_O_6_ as determined by HRESIMS data (*m*/*z* 413.1925 [M + Na]^+^, Calcd. 413.1934). The NMR spectroscopic data of compound **3** (Table 1, Table 2) indicated that it was an acetoxylated derivative of **2**, as indicated by the presence of an acetoxyl group [δ_H_ 2.09 (3H, s); δ_C_ 171.0 (C), 20.9 (CH_3_)] and an acetoxy-bearing methylene group [δ_H_ 4.62 (2H, s, H_2_-18); δ_C_ 61.9 (CH_2_, C-18)]. The attachment of the acetoxyl group to C-18 was revealed by the HMBC correlations from the acetoxy-bearing methylene protons [δ_H_ 4.62 (2H, s, H_2_-18)] to the carbonyl carbon resonating at δ_C_ 171.0 (C), two olefinic carbons C-3 and C-4 [δ_C_ 127.4 (CH) and 135.9 (C), respectively], and an aliphatic methylene carbon C-5 (δ_C_ 30.6). The relative stereochemistry of **3** was in agreement with that of **2** based on the similar NMR and NOE data. Thus, compound **3** was established as (1R*,7S*,14S*,3*E*,11*E*)-18-acetoxy-7-hydroperoxycembra-3,8(19),11,15(17)-tetraen-16,14-olide.

Compounds **1**–**3** were tested for their cytotoxicity against a panel of tumor cell lines including SGC7901 (human gastric carcinoma), A549 (human lung epithelial carcinoma), MCF7 (human breast carcinoma), HCT116 (human colonic carcinoma), and B16 (mouse melanoma). The bioassay results showed that compounds **1**–**3** possess moderate cytotoxicity against the selected tumor cell lines (Table 3).

**Table 3:** Cytotoxicity data of compounds **1**–**3**.

Compounds	IC_50_ (μg/mL)				
	SGC7901	A549	MCF7	HCT116	B16
**1**	5.3	6.1	3.8	5.2	8.6
**2**	2.7	3.2	1.2	4.5	2.1
**3**	2.3	1.8	2.9	3.4	5.6

In addition, all compounds were evaluated for the antimicrobial activity against *Staphylococcus aureus*, *S. pneumoniae*, *Pseudomonas aerugonisa*, *Saccharomyces cerevisiae*, and *Aspergillus fumigatus*. The antibiotic assay revealed that compounds **2** and **3** exhibited moderate inhibition against *Staphylococcus aureus* and *S. pneumoniae* with the inhibitory rates around 90% at 20 μg/mL, but the other compounds were weak inhibitors against the two bacterial strains. All compounds showed weak effects against the microorganisms *Pseudomonas aerugonisa*, *Saccharomyces cerevisiae*, and *Aspergillus fumigatus*.

## 3. Experimental Section

### 3.1. General Experimental Procedures

Optical rotations were determined with a PoLAAR 3005 digital polarimeter. IR spectra were obtained on a Bruker Equinox 55 spectrometer. ^1^H and ^13^C NMR and 2D NMR were recorded on a Bruker Avance 600 MHz NMR spectrometer using TMS as an internal standard. Chemical shifts (δ) were expressed in parts per million (ppm), and coupling constants (*J*) were reported in Hertz (Hz). HRESIMS data were recorded by a Thermo Scientific Q Exactive hybrid quadrupole-Orbitrap mass spectrometer. Silica gel (200–300 mesh) for column chromatography and GF_254_ silica gel for TLC was provided by Qingdao Marine Chemistry Co., Ltd. High-performance liquid chromatography (HPLC) chromatography was carried out using an Agilent 1100 series instrument equipped with a VWD G1314A detector at 210 nm and a YMC-Pack C_18_ (10 μm, 250 × 10 mm) column.

### 3.2. Animal Material

The soft coral *Lobophytum* sp. was collected from the inner coral reef at a depth of 8 m in Sanya Bay, Hainan Island of China, in November 2011, and the fresh samples were frozen immediately after collection. The specimen was identified by Dr. Xiu-Bao Li (South China Sea Institute of Oceanology, CAS, Guangzhou, China). A voucher specimen (HS201105) is deposited at the Institute of Natural Drugs Development, Wenzhou Medical College, China.

### 3.3. Extraction and Isolation

The frozen soft coral *Lobophytum* sp. (1.8 kg) was homogenized and then extracted with 95% EtOH (4 × 3 L) at room temperature. The EtOH extract (116.9 g) was partitioned between H_2_O and EtOAc. The EtOAc fraction (21.9 g) was subjected to silica gel (200–300 mesh) column chromatography, and was eluted with a gradient of petroleum ether (PE)/EtOAc (10:1, 5:1, 2:1, 1:1) to obtain seven fractions (F1–F7). F3 (0.5 g) was fractioned on Sephadex LH-20 (70 × 2.5 cm, eluted with CH_2_Cl_2_/MeOH 1:1) to afford four subfractions (F3A–F3D). The subfraction F3C (73 mg) was further separated on reversed-phase semi-preparative HPLC with MeOH/H_2_O (95:5) as a mobile phase to obtain **4** (18.8 mg) and **14** (3.0 mg). F5 (3.7 g) was subjected to silica gel (200–300 mesh) column eluting with PE/CH_2_Cl_2_ (1:1) to afford six subfractions (F5A–F5F). The subfraction F5C (324.6 mg) was separated on silica gel (200–300 mesh) column eluting with PE/Acetone (10:1), and further purified by an ODS column (C_18_, 25 × 2 cm, eluted with MeOH/H_2_O 85:15) to obtain **12** (20.0 mg). The subfraction F5E (1.1 g) was subjected to Sephadex LH-20 column eluting with CH_2_Cl_2_/MeOH (1:1), and further purified by HPLC (MeOH/H_2_O, 80:20) to afford **1** (4.9 mg), **2** (2.3 mg), **3** (5.8 mg), **8** (14.9 mg), **9** (21.2 mg), and **13** (6.2 mg). F6 (5.1 g) was separated on silica gel (200–300 mesh) column, eluted with CH_2_Cl_2_/EtOAc (30:1) to afford four subfractions (F6A–F6D). The subfraction F6B (0.9 g) was subjected to ODS column (C_18_, 25 × 2 cm, eluted with MeOH/H_2_O 80:20), and further purified by HPLC (MeOH/H_2_O, 80:20) to afford **11** (14.2 mg), **5** (10.0 mg), **6** (9.6 mg), and **7** (10.6 mg). Compound **10** (16.9 mg) was obtained from F6C (0.5 g) by the same separation process as that for F6B. 

(1R*,3R*,4R*,14R*,7*E*,11*E*)-3,4-epoxycembra-7,11,15(17)-trien-16,14-olide (**1**), obtained as colorless oil; [α]_D_^25^ +133.4 (*c* 0.17, CHCl_3_); IR (KBr) ν_max_ 2903, 1759, 1660, 1441, 1342, 1270, 1155, 1123, 994 cm^−1^; ^1^H and ^13^C NMR data, see Table 1, Table 2; HRESIMS (*m/z*) 317.2105 [M + H]^+^ (Calcd. for C_2__0_H_29_O_3_, 317.2111).

(1R*,7S*,14S*,3*E*,11*E*)-7-hydroperoxycembra-3,8(19),11,15(17)-tetraen-16,14-olide (**2**), obtained as colorless oil; [α]_D_^25^ +150.9 (*c* 0.14, CHCl_3_); IR (KBr) ν_max_ 3411, 2962, 2930, 2865, 1762, 1659, 1643, 1272, 1169, 1080 cm^−1^; ^1^H and ^13^C NMR data, see Table 1, Table 2; HRESIMS (*m/z*) 355.1876 [M + Na]^+^ (Calcd. for C_2__0_H_28_O_4_Na, 355.1880).

(1R*,7S*,14S*,3*E*,11*E*)-18-acetoxy-7-hydroperoxycembra-3,8(19),11,15(17)-tetraen-16,14-olide (**3**), obtained as colorless oil; [α]_D_^25^ +214.4 (*c* 0.10, CHCl_3_); IR (KBr) ν_max_ 3420, 2964, 2925, 2863, 1760, 1745, 1660, 1235, 1095 cm^−1^; ^1^H and ^13^C NMR data, see Table 1, Table 2; HRESIMS (*m/z*) 413.1925 [M + Na]^+^ (Calcd. for C_2__2_H_30_O_6_Na, 413.1934).

### 3.4. Cytotoxicity Assay

The cytotoxic properties of the isolated compounds were tested *in vitro* using tumor cell lines including SGC7901 (human gastric carcinoma), A549 (human lung epithelial carcinoma), MCF7 (human breast carcinoma), HCT116 (human colonic carcinoma), and B16 (mouse melanoma) tumor cells by a modification of the MTT colorimetric method according to a previously described procedure [26,27]. The cell lines were purchased from the Cell Resource Center of Shanghai Institute of Biological Sciences, CAS.

### 3.5. Antibiotic Assay

Antimicrobial bioassays were conducted in triplicate according to the method recommended by the National Center for Clinical Laboratory Standards (NCCLS) [28]. The bacterial strains *Staphylococcus aureus*, *S. pneumoniae*, and *Pseudomonas aerugonisa* were grown on Mueller-Hinton agar. The yeast, *Saccharomyces cerevisiae*, was grown on Sabouraud dextrose agar, and the fungus, *Aspergillus fumigatus*, was grown on potato dextrose agar. Targeted microbes (3–4 colonies) were prepared from broth culture (bacteria: 37 °C for 24 h; fungus: 28 °C for 48 h), and the final spore suspensions of bacteria (in MHB medium), yeast (in SDB medium), and fungus (in PDB medium) were 10^6^ and 10^5^ cells/mL and 10^4^ mycelial fragments/mL, respectively. Testing compounds (10 mg/mL as stock solution in DMSO and serial dilutions) were transferred to a 96-well clear plate in triplicate, and the suspension of the test microorganisms were added to each well (200 μL) (antimicrobial peptide AMP, streptomycin, and fluconazole were used as positive controls). After incubation, the absorbance at 595 nm was measured with a microplate reader (TECANT), and the inhibition rate was calculated and plotted *versus* test concentrations.

## 4. Conclusions

Three new α-methylene-γ-lactone-containing cembranoids, namely (1R*,3R*,4R*,14R*,7*E*,11*E*)-3,4-epoxycembra-7,11,15(17)-trien-16,14-olide (**1**), (1R*,7S*,14S*,3*E*,11*E*)-7-hydroperoxycembra-3,8(19),11,15(17)-tetraen-16,14-olide (**2**), and (1R*,7S*,14S*,3*E*,11*E*)-18-acetoxy-7-hydroperoxycembra-3,8(19),11,15(17)-tetraen-16,14-olide (**3**), along with eleven known analogues **4**–**14**, were isolated from the South China Sea soft coral *Lobophytum* sp. Compounds **2** and **3** contain a rare hydroperoxyl group at C-7. The isolation of compounds **1**–**3** constitutes a new addition to the molecular diversity of cembrane-type diterpenoids. In addition, compounds **1**–**3** were found to show moderate cytotoxic activity against the selected tumor cell lines including SGC7901 (human gastric carcinoma), A549 (human lung epithelial carcinoma), MCF7 (human breast carcinoma), HCT116 (human colonic carcinoma), and B16 (mouse melanoma) with IC_50_ values ranged from 1.2 to 8.6 μg/mL. Compounds **2** and **3** displayed moderate inhibition against the bacteria *S. aureus* and *S. pneumoniae* with inhibitory rates of around 90% at 20 μg/mL, suggesting them to be promising lead structures for antibiotics. Further studies should be conducted to elucidate the antibacterial mechanism of **2** and **3**, as well as to understand the ecological roles of these metabolites in the life cycle of the soft coral.

## Supplementary Files

Supplementary File 1 Supplementary Information (PDF, 374 KB)
