# Supplementary material for: Cytotoxic and Antibacterial Cembranoids from a South China Sea Soft Coral, Lobophytum sp
Source: Mar Drugs. 2013 Apr 3;11(4):1162–72. doi: 10.3390/md11041162 (PMC3705396; doi:10.3390/md11041162)
Supplement: Supplementary File 1 — Supplementary Information (PDF, 374 KB) [file marinedrugs-11-01162-s001.pdf]

Supplementary Information

Figure S1. ^{1}H NMR spectrum (600 MHz) of compound **1** in $CDCl_3$. 2
Figure S2. ^{13}C NMR spectrum (150 MHz) of compound **1** in $CDCl_3$. 2
Figure S3. HMQC spectrum (600 MHz) of compound **1** in $CDCl_3$. 3
Figure S4. HMBC spectrum (600 MHz) of compound **1** in $CDCl_3$. 3
Figure S5. COSY spectrum (600 MHz) of compound **1** in $CDCl_3$. 4
Figure S6. NOESY spectrum (600 MHz) of compound **1** in $CDCl_3$. 4
Figure S7. HRESIMS spectrum of compound **1**. 5
Figure S8. ^{1}H NMR spectrum (600 MHz) of compound **2** in $CDCl_3$. 5
Figure S9. ^{13}C NMR spectrum (150 MHz) of compound **2** in $CDCl_3$. 6
Figure S10. HMQC spectrum (600 MHz) of compound **2** in $CDCl_3$. 6
Figure S11. HMBC spectrum (600 MHz) of compound **2** in $CDCl_3$. 7
Figure S12. COSY spectrum (600 MHz) of compound **2** in $CDCl_3$. 7
Figure S13. NOESY spectrum (600 MHz) of compound **2** in $CDCl_3$. 8
Figure S14. HRESIMS spectrum of compound **2**. 8
Figure S15. ^{1}H NMR spectrum (600 MHz) of compound **3** in $CDCl_3$. 9
Figure S16. ^{13}C NMR spectrum (150 MHz) of compound **3** in $CDCl_3$. 9
Figure S17. HMQC spectrum (600 MHz) of compound **3** in $CDCl_3$. 10
Figure S18. HMBC spectrum (600 MHz) of compound **3** in $CDCl_3$. 10
Figure S19. COSY spectrum (600 MHz) of compound **3** in $CDCl_3$. 11
Figure S20. NOESY spectrum (600 MHz) of compound **3** in $CDCl_3$. 11
Figure S21. HRESIMS spectrum of compound **3**. 12

Figure S1. ^{1}H NMR spectrum (600 MHz) of compound **1** in $CDCl_3$.

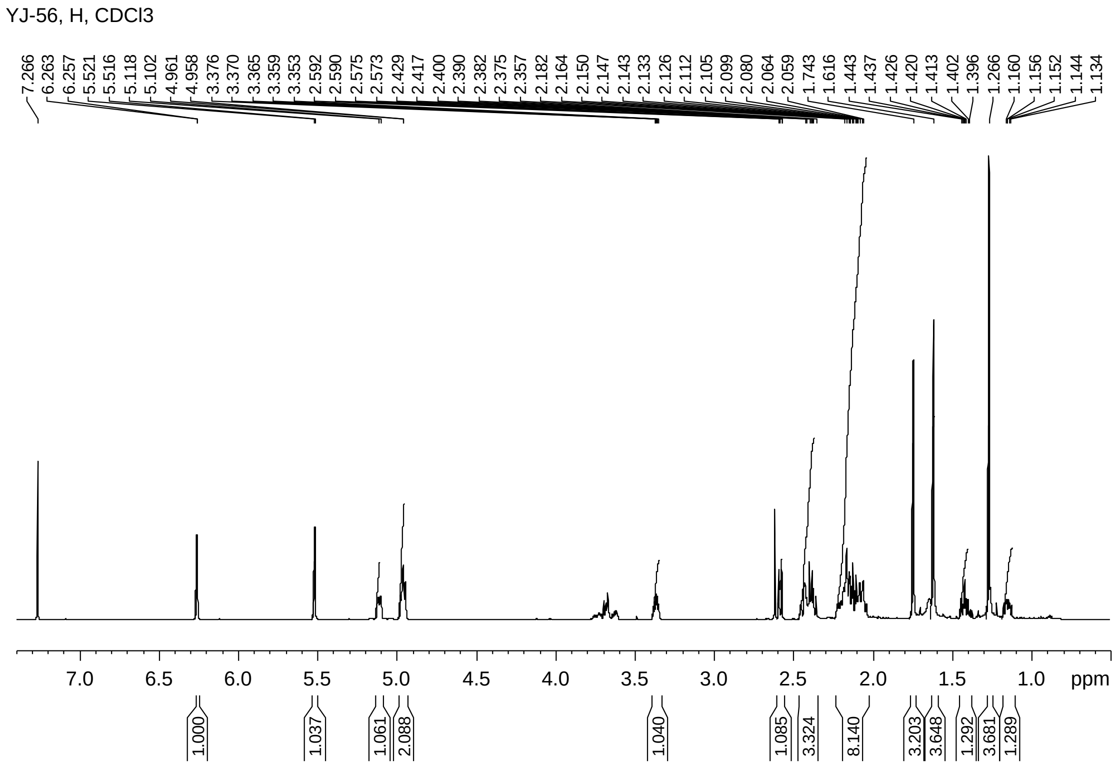

Figure S2. ^{13}C NMR spectrum (150 MHz) of compound **1** in $CDCl_3$.

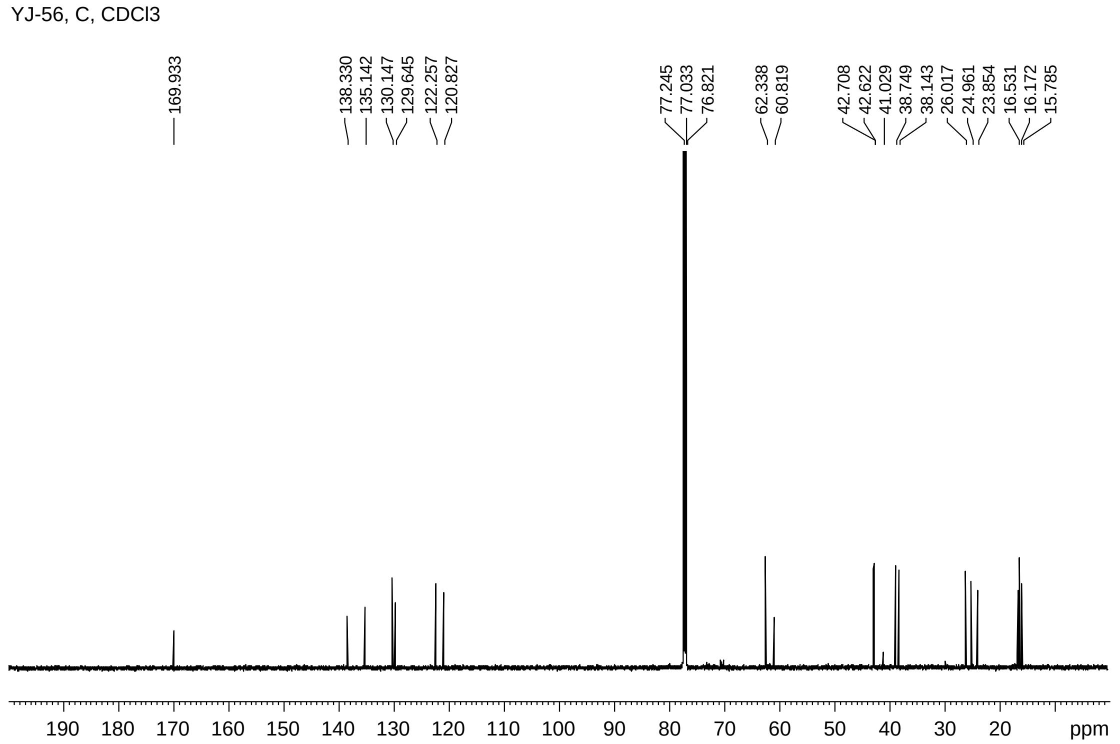

Figure S3. HMQC spectrum (600 MHz) of compound **1** in $CDCl_3$.

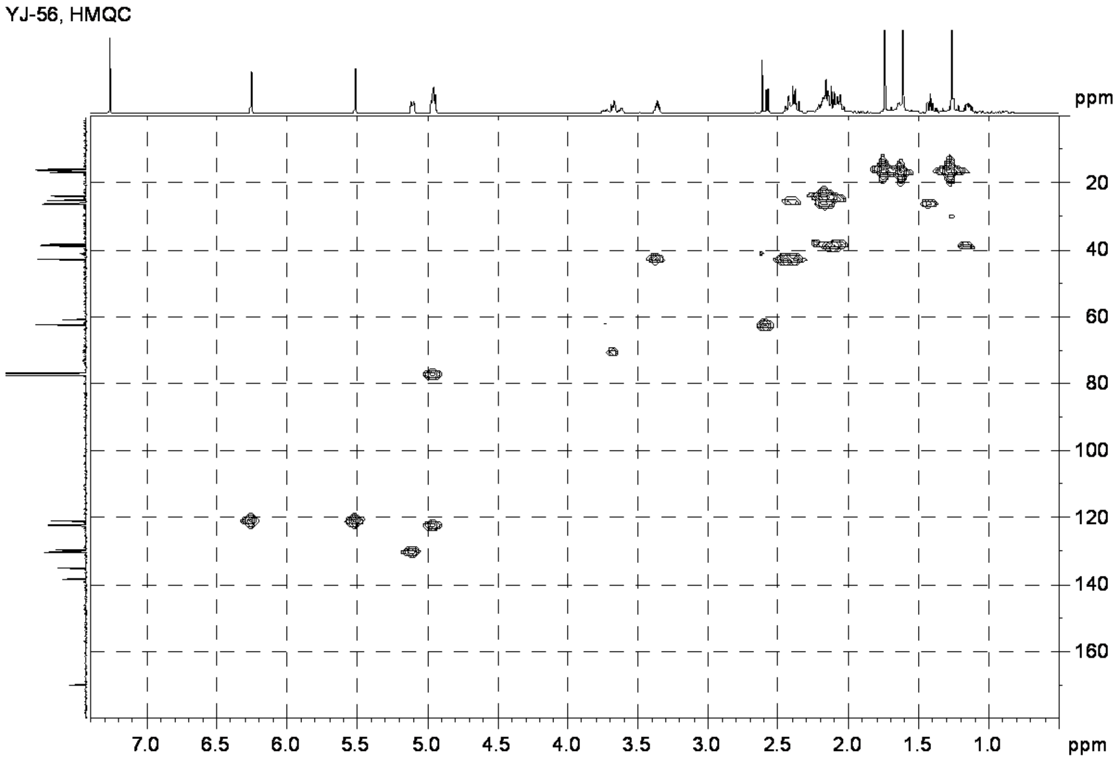

Figure S4. HMBC spectrum (600 MHz) of compound **1** in $CDCl_3$.

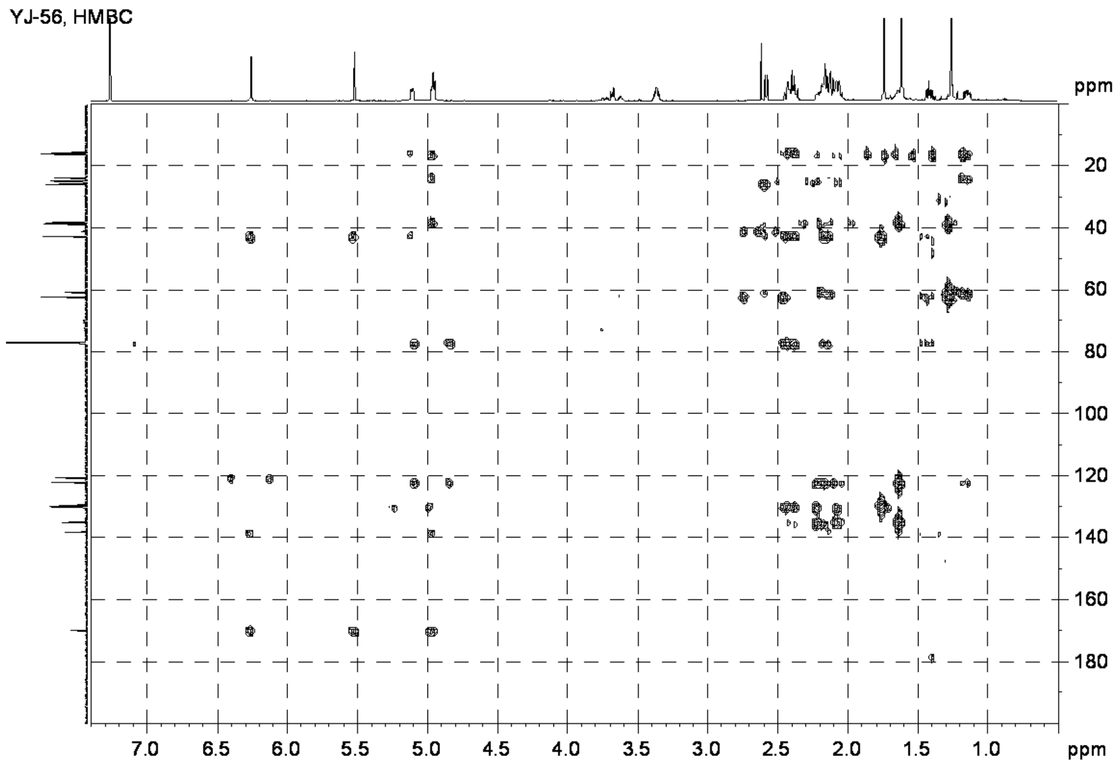

Figure S5. COSY spectrum (600 MHz) of compound **1** in $CDCl_3$.

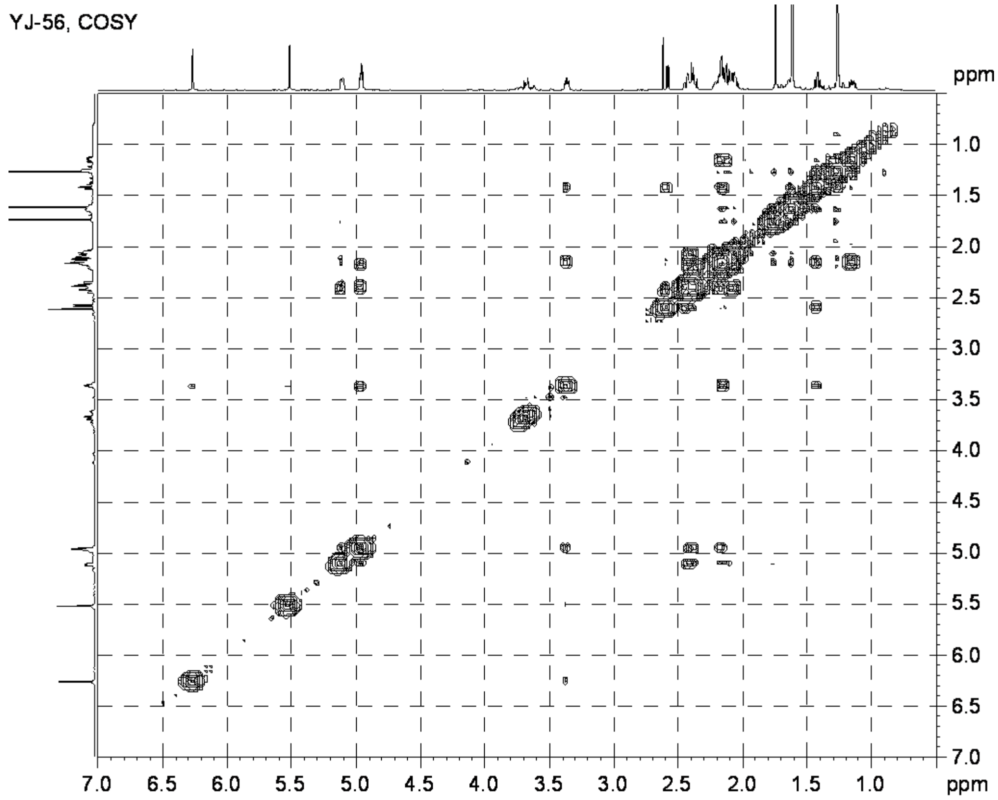

Figure S6. NOESY spectrum (600 MHz) of compound **1** in $CDCl_3$.

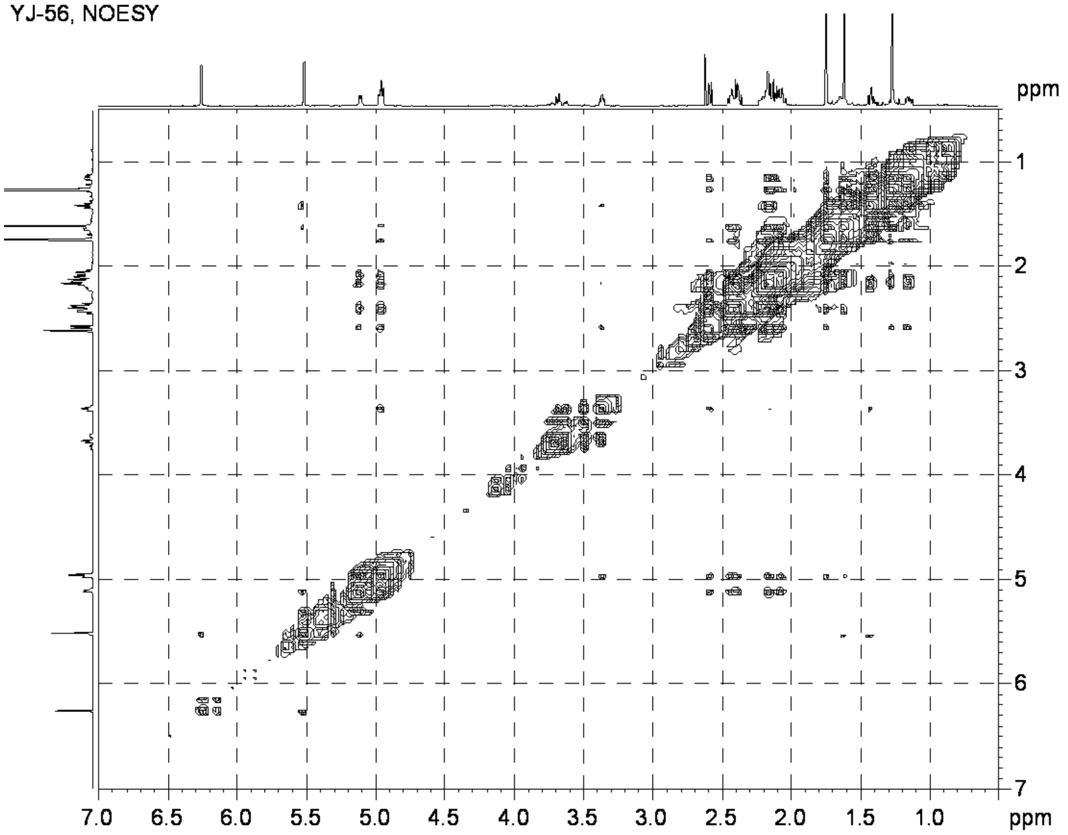

Figure S7. HRESIMS spectrum of compound **1**.

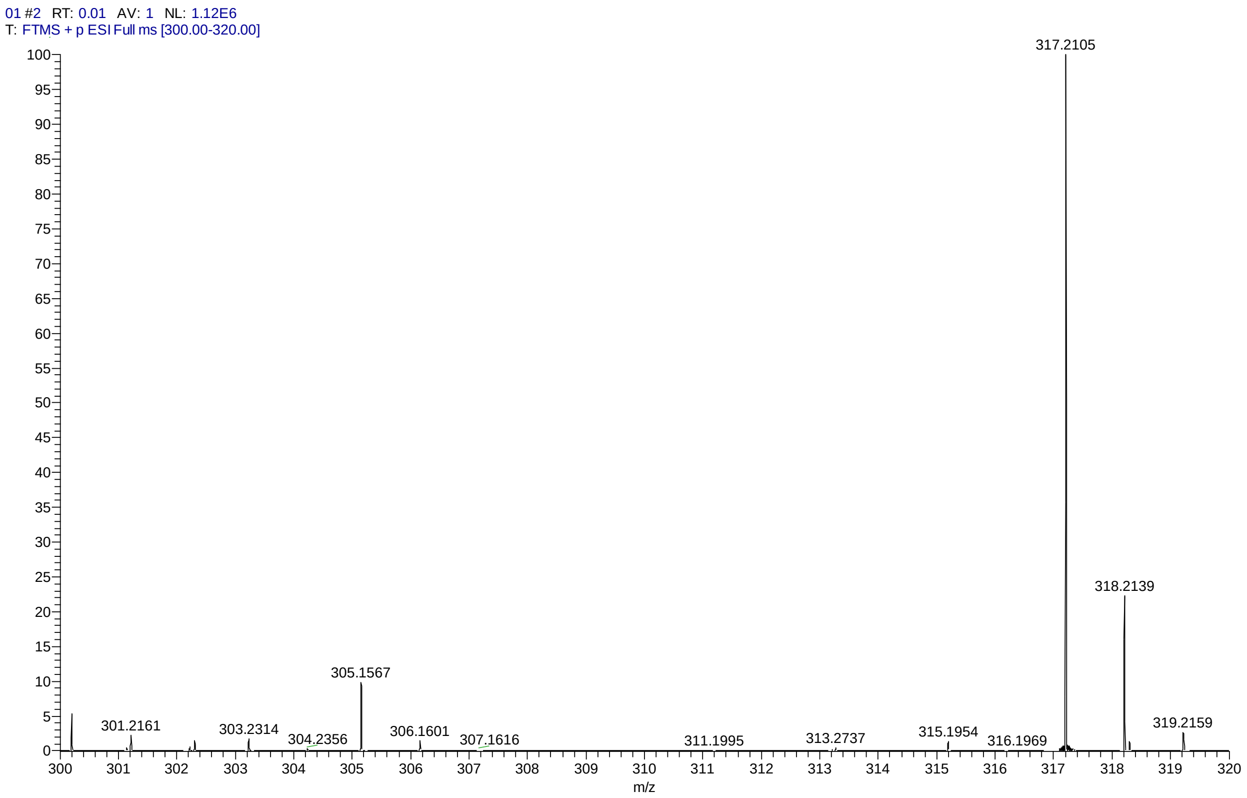

Figure S8. ^{1}H NMR spectrum (600 MHz) of compound **2** in $CDCl_3$.

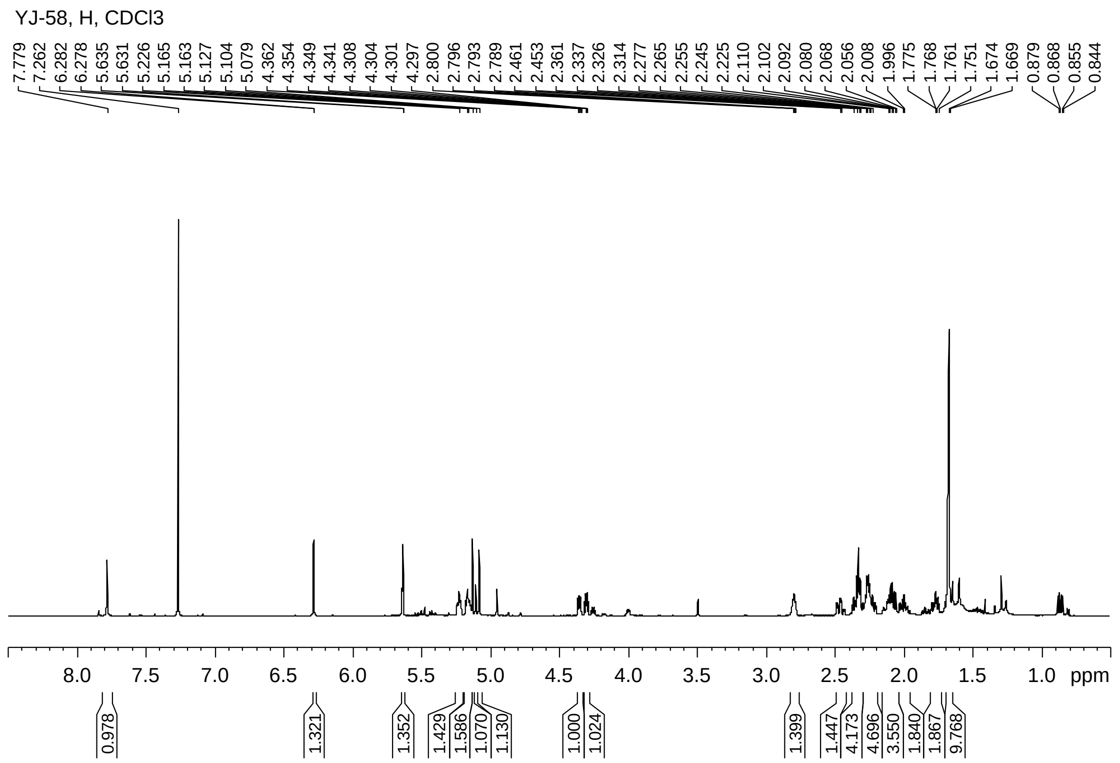

Figure S9. ^{13}C NMR spectrum (150 MHz) of compound **2** in $CDCl_3$.

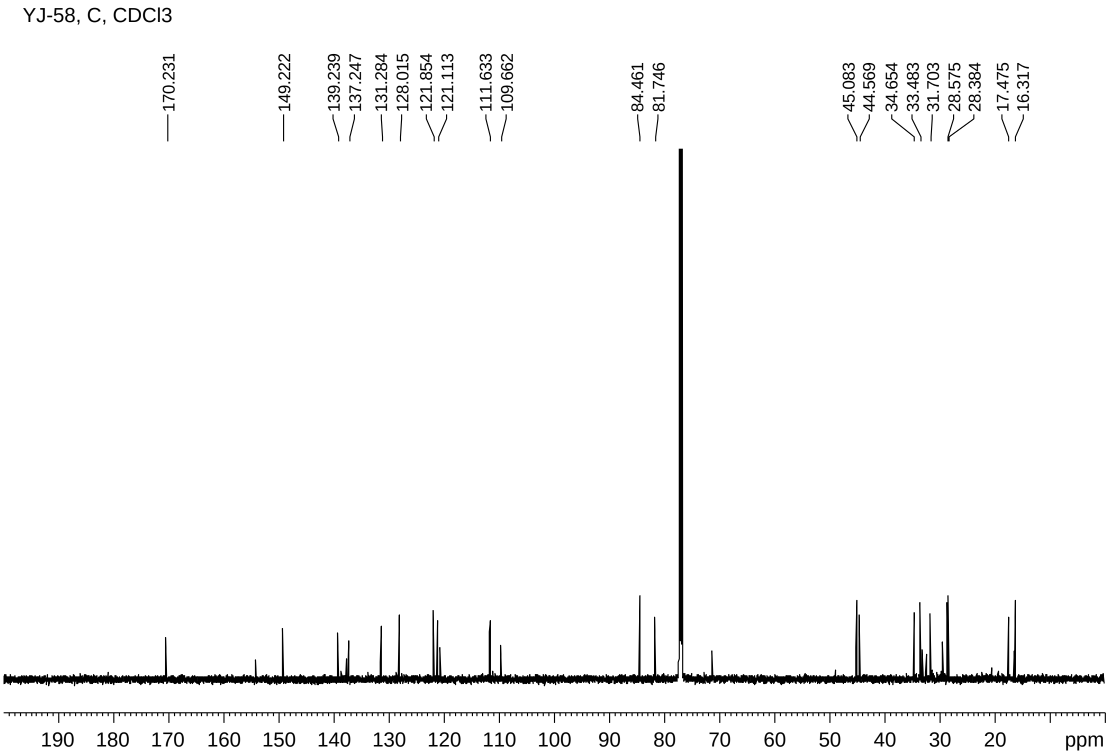

Figure S10. HMQC spectrum (600 MHz) of compound **2** in $CDCl_3$.

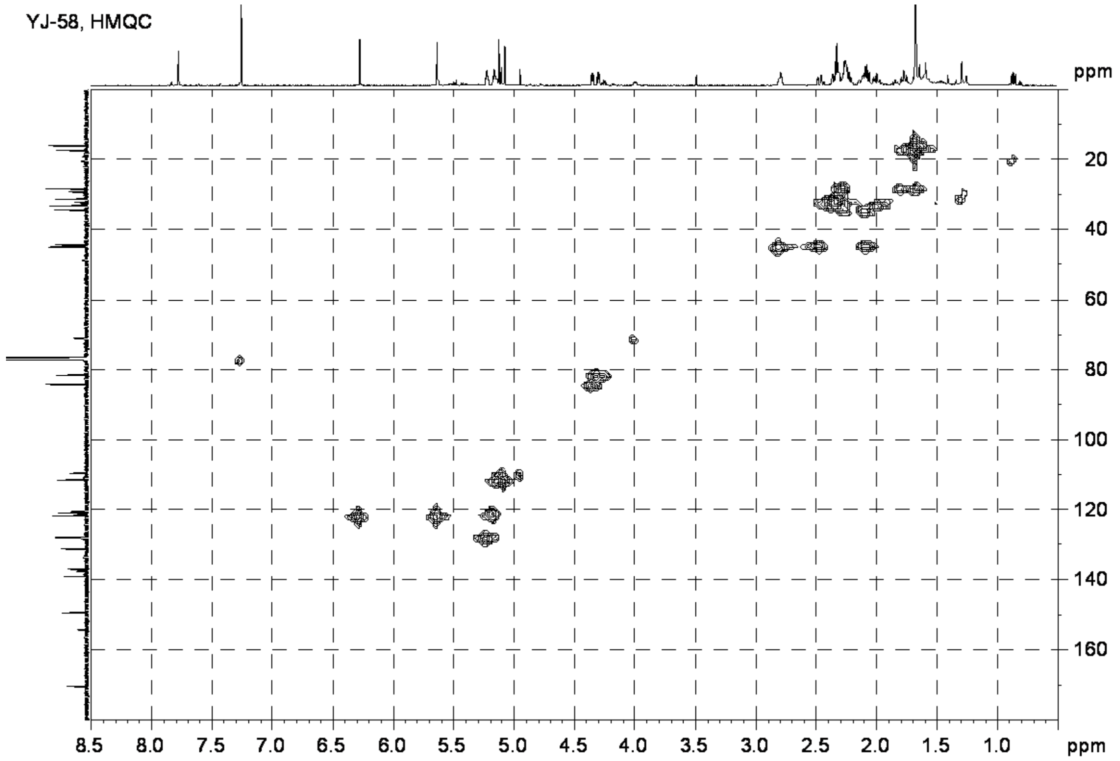

Figure S11. HMBC spectrum (600 MHz) of compound **2** in $CDCl_3$.

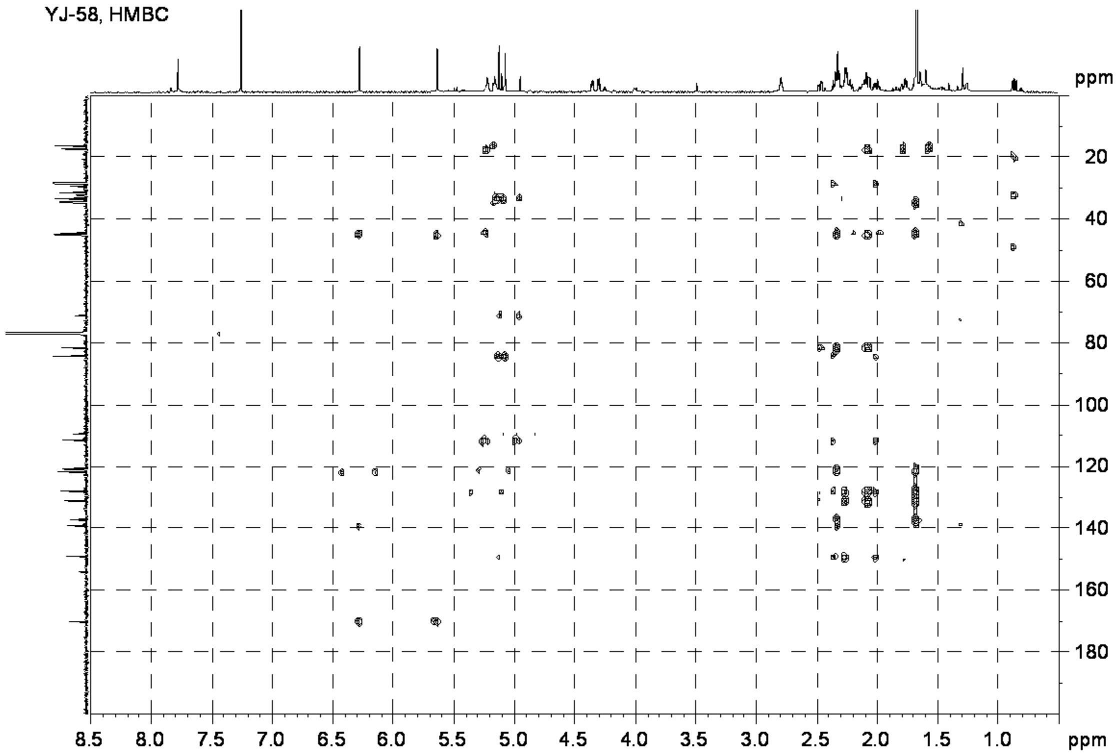

Figure S12. COSY spectrum (600 MHz) of compound **2** in $CDCl_3$.

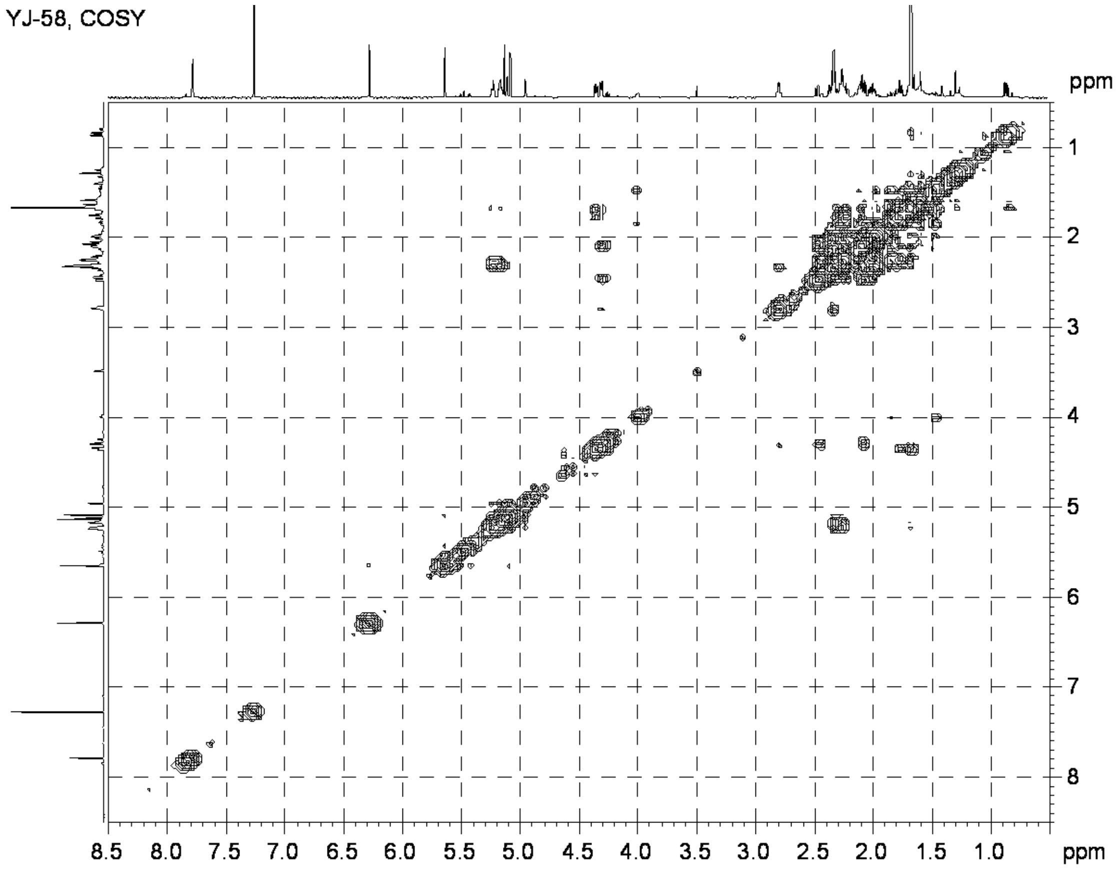

Figure S13. NOESY spectrum (600 MHz) of compound **2** in $CDCl_3$.

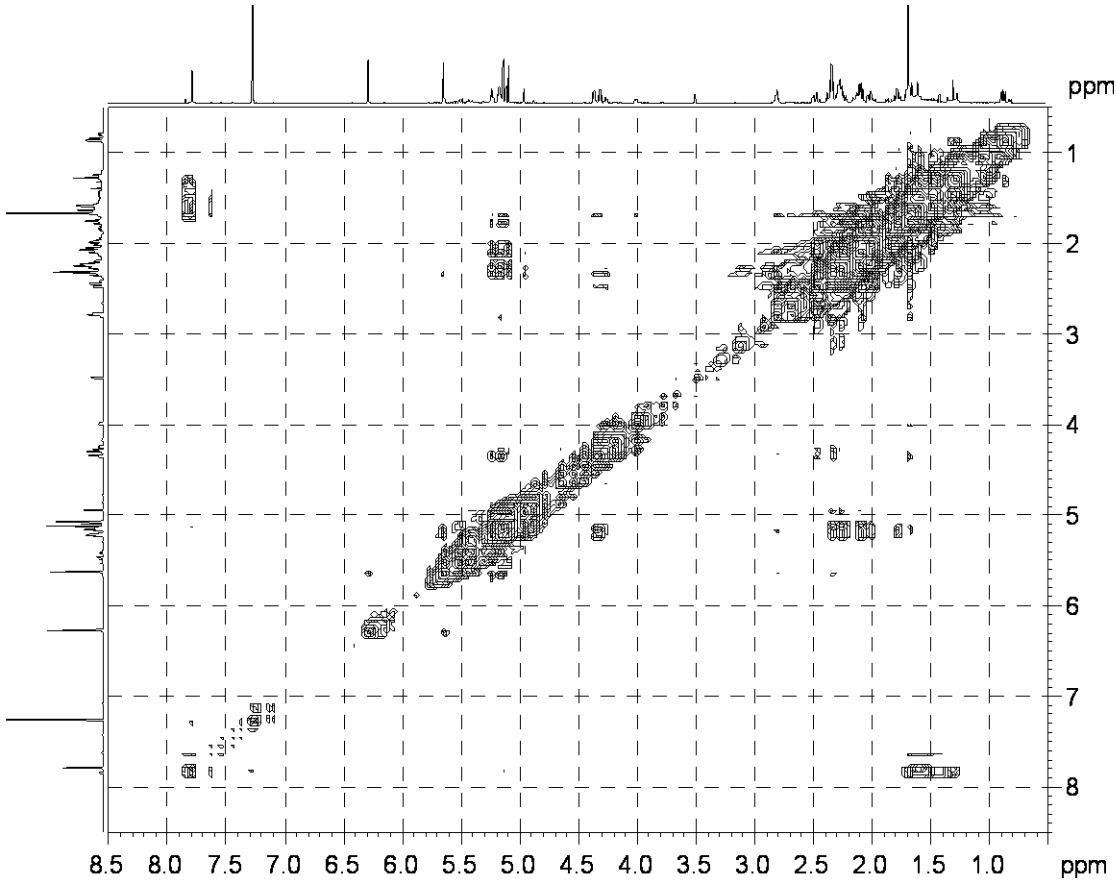

Figure S14. HRESIMS spectrum of compound **2**.

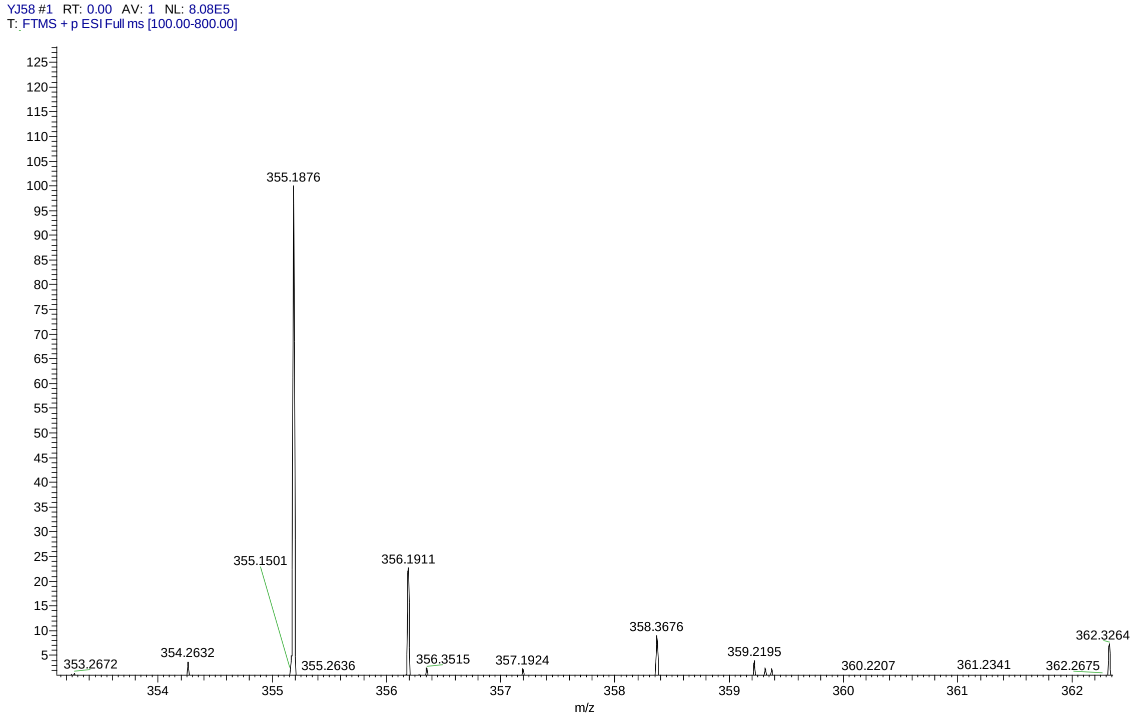

Figure S15. ^{1}H NMR spectrum (600 MHz) of compound **3** in $CDCl_3$.

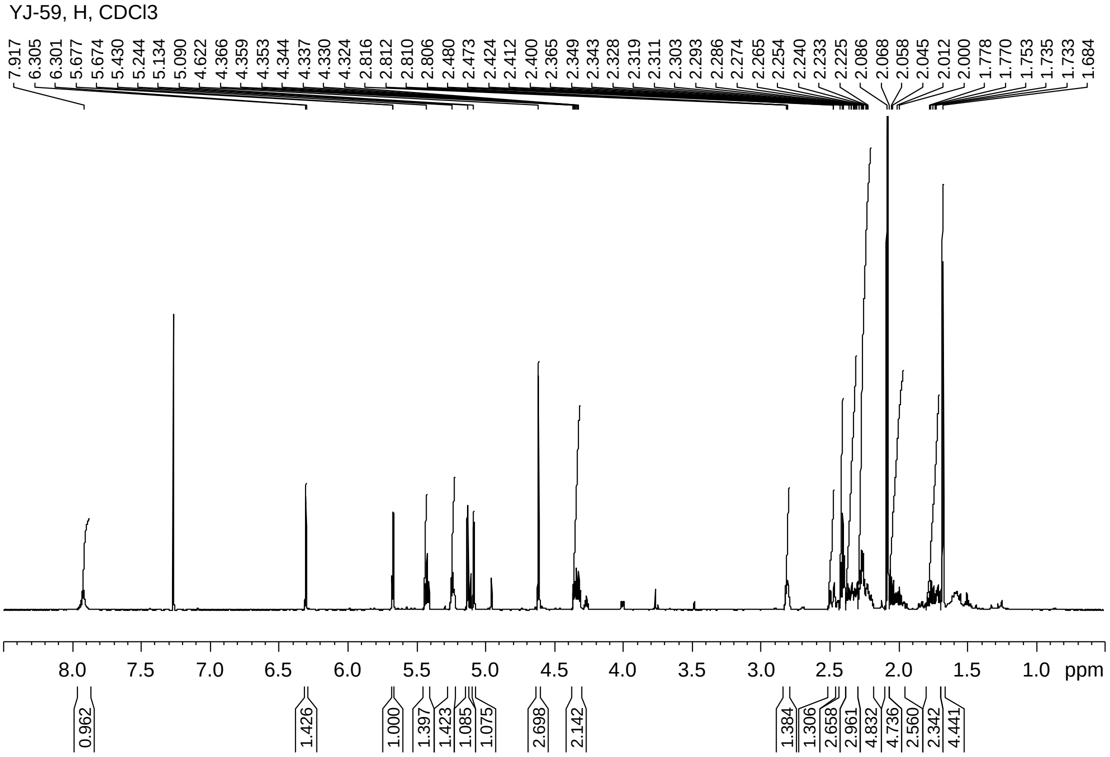

Figure S16. ^{13}C NMR spectrum (150 MHz) of compound **3** in $CDCl_3$.

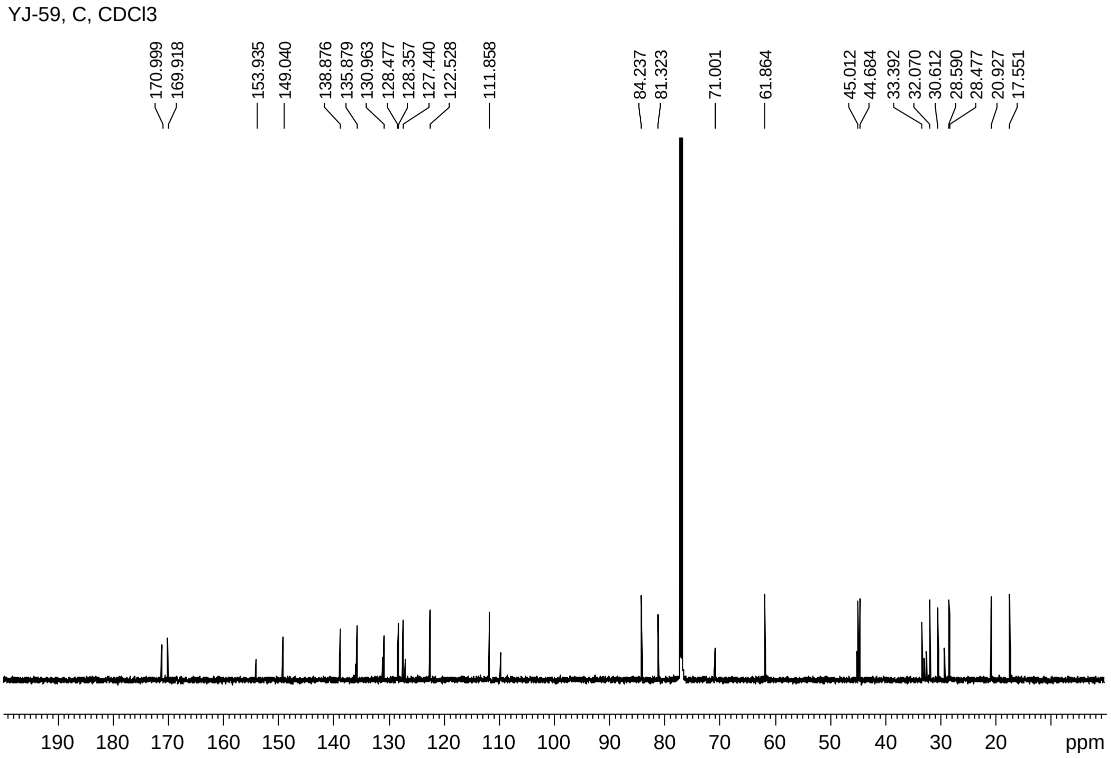

Figure S17. HMQC spectrum (600 MHz) of compound **3** in $CDCl_3$.

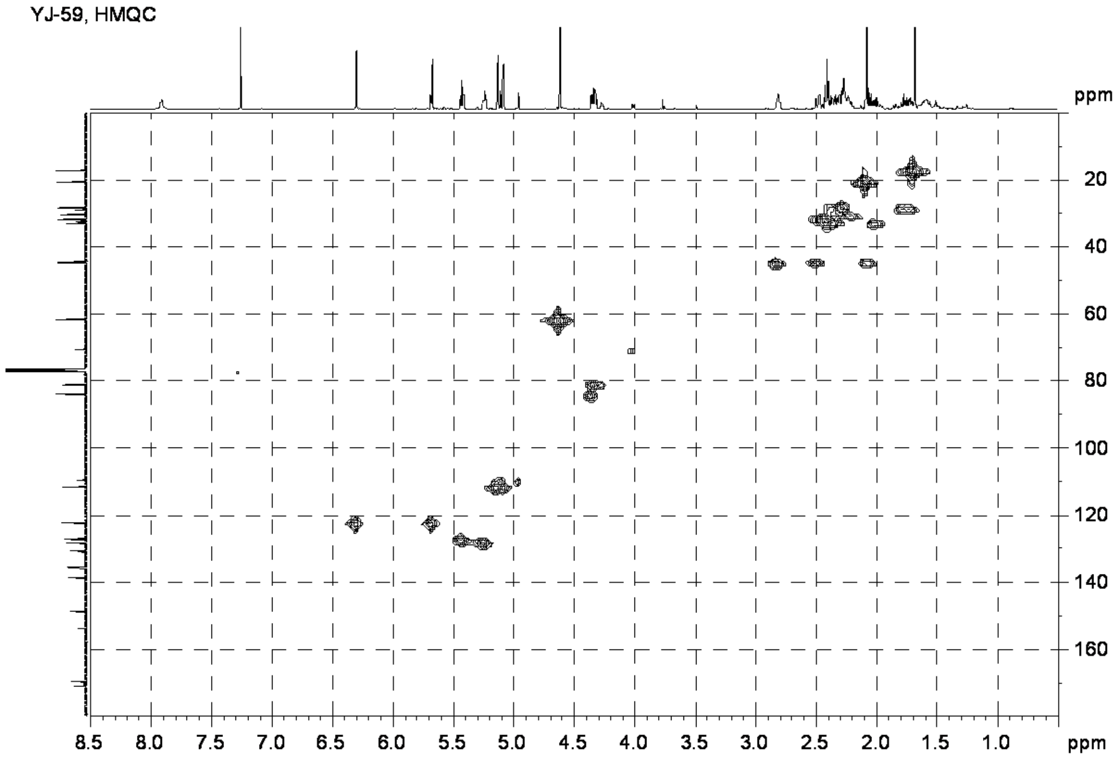

Figure S18. HMBC spectrum (600 MHz) of compound **3** in $CDCl_3$.

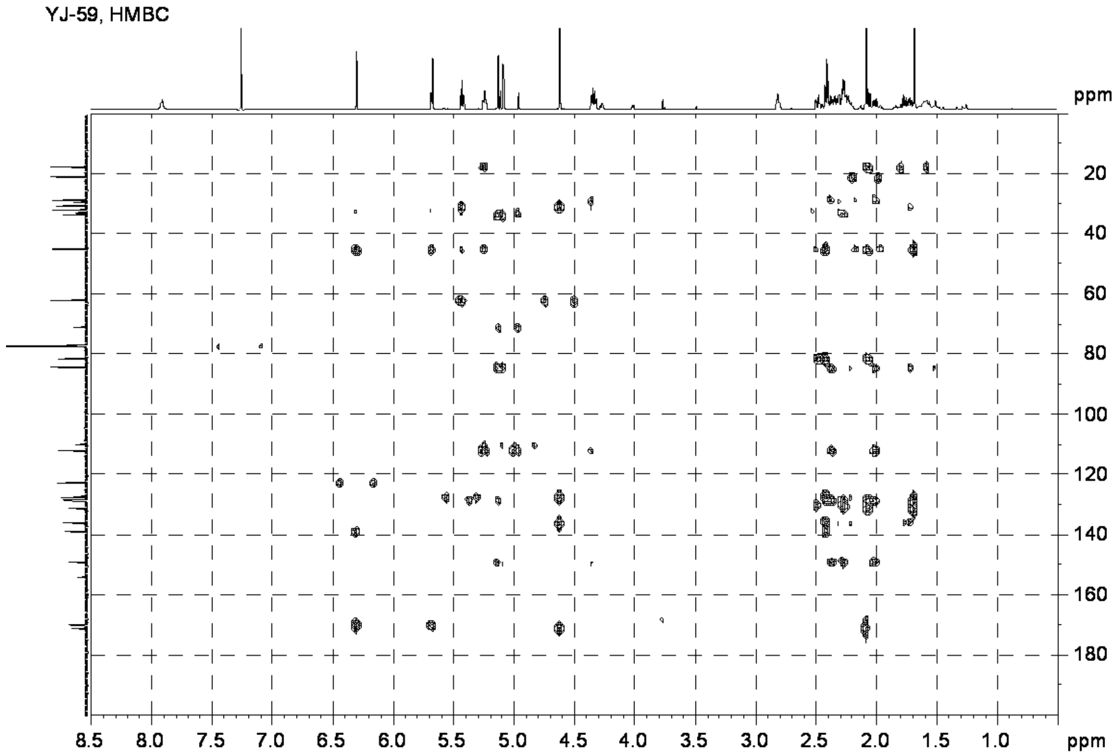

Figure S19. COSY spectrum (600 MHz) of compound **3** in $CDCl_3$.

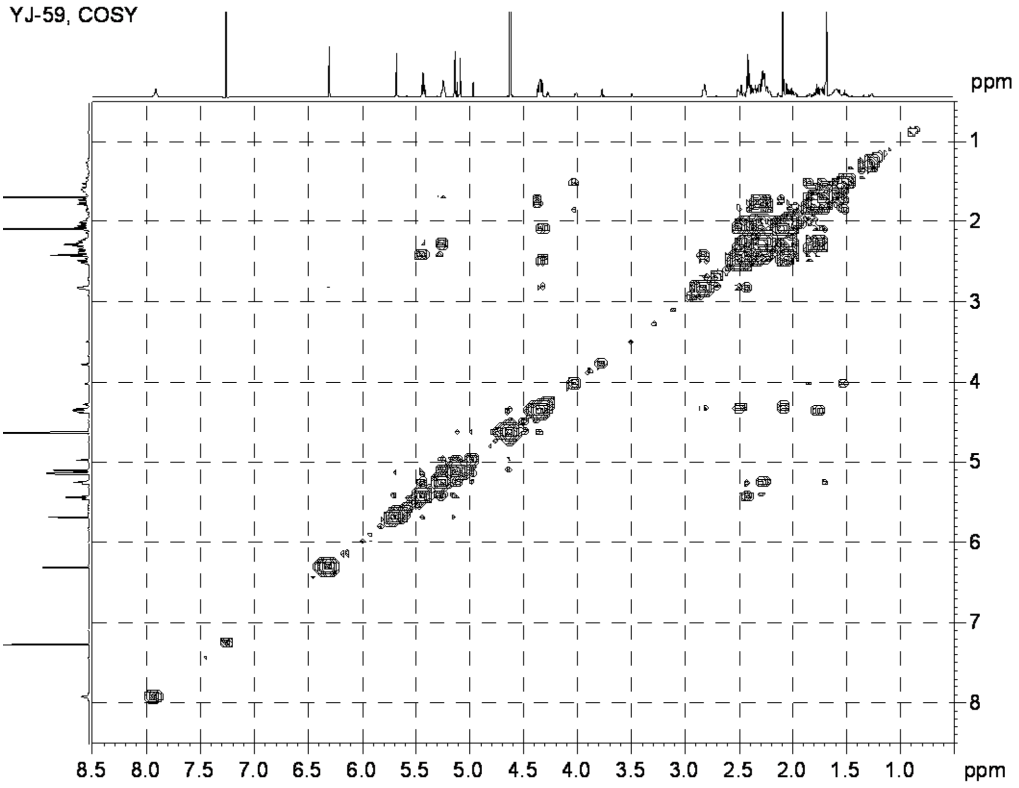

Figure S20. NOESY spectrum (600 MHz) of compound **3** in $CDCl_3$.

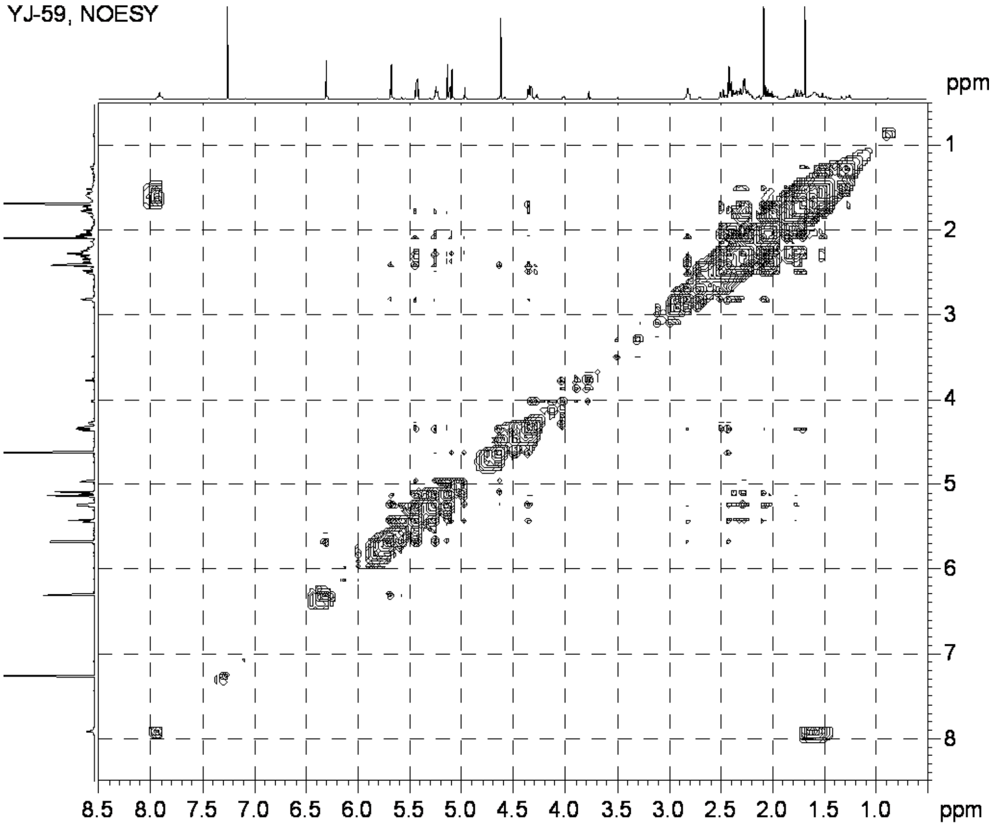

Figure S21. HRESIMS spectrum of compound **3**.

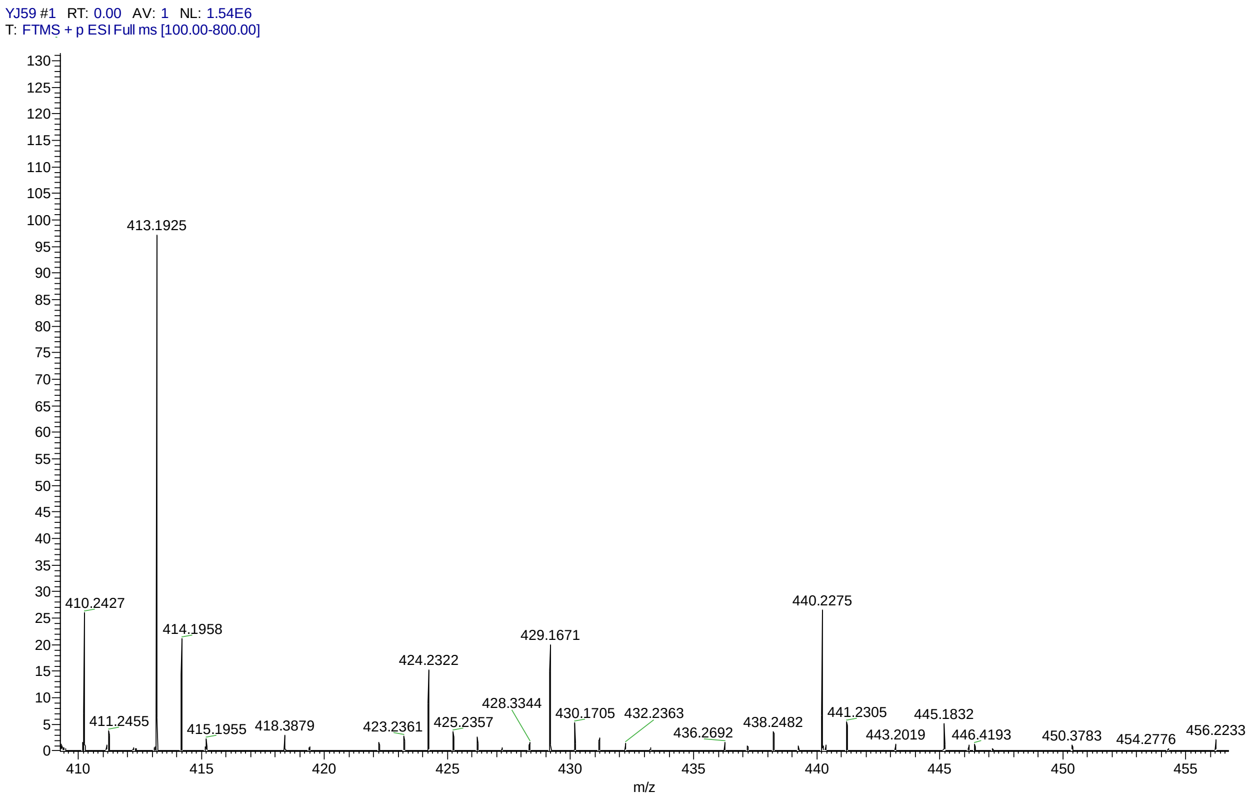

© 2013 by the authors; licensee MDPI, Basel, Switzerland. This article is an open access article distributed under the terms and conditions of the Creative Commons Attribution license (http://creativecommons.org/licenses/by/3.0/).
